# An Easy New Method for Reducing Anterior Shoulder Dislocations: The Lateral Position Technique

**DOI:** 10.7759/cureus.88803

**Published:** 2025-07-26

**Authors:** Koichiro Makihara, Nobuhiko Makihara, Yasuhiko Takegami

**Affiliations:** 1 Department of Orthopaedic Surgery, Shizuoka Saiseikai General Hospital, Shizuoka, JPN; 2 Department of Orthopaedic Surgery, Makihara Orthopaedic Clinic, Kota, JPN; 3 Department of Orthopaedic Surgery, Nagoya University Graduate School of Medicine, Nagoya, JPN

**Keywords:** acute anterior dislocation, emergency reduction techniques, lateral position maneuver, shoulder dislocation, traction methods

## Abstract

Background

Anterior shoulder dislocation is the most common dislocation that occurs in the human body, and reduction can still be a major challenge to the physician.

Objectives

To develop a new reduction technique called the lateral position maneuver (LPM) method (also known as the Makihara method), which is performed in a lateral position by a single physician without sedation.

Methods

Eighty-five patients with clinically and radiographically proven acute anterior shoulder dislocations were included. The LPM method was used for all the patients included in the study. The inclusion criterion was a traumatic anterior dislocation of the shoulder that presented within 24 hours of injury, including those with a greater tuberosity fracture or Bankart lesion. Patients with multiple traumas, Neers three- or four-part fractures, insufficient clinical data, neuropathy, and dislocations repaired by other methods were excluded. Ultimately, 13 shoulders (13 patients) were included in the study.

Results

Numerous reduction techniques for anterior glenohumeral joints exist, and the success rate for first-time anterior shoulder dislocations ranges from 70% to 90%. Most of these techniques required assistance and sedation during the procedure; however, our technique is easy to perform as we were able to achieve a closed reduction of acute anterior dislocation, requiring only a single physician to perform it without the use of sedation.

Conclusions

This study showed that there were no complications from the use of the LPM method (Makihara method) and that it is useful. The number of patients included in this study was small, but further additional studies are needed to confirm its efficacy.

## Introduction

The glenohumeral joint is the most mobile joint in the body [1]. Anterior dislocation of this joint accounts for more than 50% of dislocations in the body [2]. The incidence of dislocations varies between 23.91 and 23.12 per 100,000 person-years, with a higher incidence in young men (98.3 per 100,000 person-years) [3]. Various methods have been described for the reduction of shoulder dislocation, such as the external rotation, Milch, and Stimson methods. In the external rotation method, reduction is achieved by gradually externally rotating the arm. The Milch technique involves additional abduction to facilitate reduction. In the Stimson method, reduction is performed by applying traction using a weight [2,4-7]. In support of an easy method for reducing anterior shoulder dislocation by junior emergency medicine residents, Yuen et al. reported an overall success rate of 87.5% for the Spaso technique in anterior dislocation of the shoulder joint, which is simple, effective, and can be performed by a single operator [8]. The Milch technique involves shoulder abduction and external rotation [9], and O’Connor et al. studied the effectiveness of this technique [10]. It is difficult to ensure successful reduction without anesthesia using any method. Reduction of anterior shoulder dislocation remains a major challenge for physicians, especially in young, muscular, non-sedated patients [11]. The Eskimo technique was introduced in the past as a method for adjusting the patient in the lateral decubitus position without sedation; however, it requires two physicians during the procedure. In the Eskimo technique, the patient is positioned laterally, with the affected arm fully extended. One operator applies upward traction on the arm, while a second operator assists the reduction by pressing on the humeral head [12]. We developed a novel method called the lateral position maneuver (LPM, Makihara method), which enables closed reduction in the lateral decubitus position without sedation and with only a single operator. The LPM combines the gravitational benefits of the Stimson method with the controlled abduction and external rotation principles of the Milch method. This study aims to introduce the LPM technique and to evaluate its feasibility and clinical outcomes through a retrospective case series.

## Materials and methods

Study design and ethical approval

This was a retrospective observational case series conducted at a single institution between April 2016 and March 2019. The study was approved by the institutional ethics committee (IRB Approval No. 2020-0564-7), and informed consent was obtained from all participants. No sedation or anesthesia was used during the procedures.

Patients

Between April 2016 and March 2019, 85 patients with clinically and radiographically proven acute anterior shoulder dislocations were included. The inclusion criterion was a traumatic anterior dislocation of the shoulder that presented within 24 hours of injury, including those with a greater tuberosity fracture or Bankart lesion. We excluded patients with multiple traumas, Neers three- or four-part fractures, patients with insufficient clinical data, patients with neuropathy, and patients with dislocations reduced by other methods. Ultimately, 13 shoulders (13 patients) were included in the study. The LPM method was used for all the patients included in the study.

Sample size and statistical considerations

This study was designed as a retrospective case series focusing on the feasibility and safety of the newly developed lateral position maneuver (LPM). The sample size of 13 was not determined based on a priori statistical power calculation but rather reflects the number of eligible cases that met the inclusion criteria over a three-year period at a single institution.

Given the exploratory nature of this study and the lack of a control group, no formal hypothesis testing or comparative statistical analyses were conducted. Therefore, statistical power analysis was not applicable. The results are descriptive in nature, intended to generate hypotheses and inform the design of future comparative studies with adequate power.

Inclusion and exclusion criteria

Patients with acute traumatic anterior shoulder dislocations presenting within 24 hours of injury were included. All 13 cases were diagnosed based on both plain radiographs and computed tomography (CT) scans. Patients with greater tuberosity fractures or Bankart lesions were not excluded. Exclusion criteria included multiple trauma, Neer three- or four-part fractures, incomplete clinical data, neuropathy, or those treated with other reduction methods.

LPM procedure 

We developed an LPM based on a conventional traction method. The procedure can be performed by a single physician. The main difference between this method and the conventional traction method is the patient's position. While conventional traction is performed in the sitting or supine position, this method is performed in the lateral decubitus position. The patient lies on a bed with the affected side facing upward. The physician positions one of his/her elbows under the patient's flexed elbow and securely holds the patient's hand. The other hand firmly grasps the patient's wrist. Then, the physician gradually abducts the shoulder joint using both hands until reaching a 90° angle. After the abduction, the surgeon gradually lifts the patient's upper body upward while maintaining the arms’ position until the patient's upper body is slightly lifted off the bed. After confirming that the body was lifted, the patient’s upper limb was slowly rotated externally. If the first attempt fails, the procedure is repeated. 

**Figure 1:**
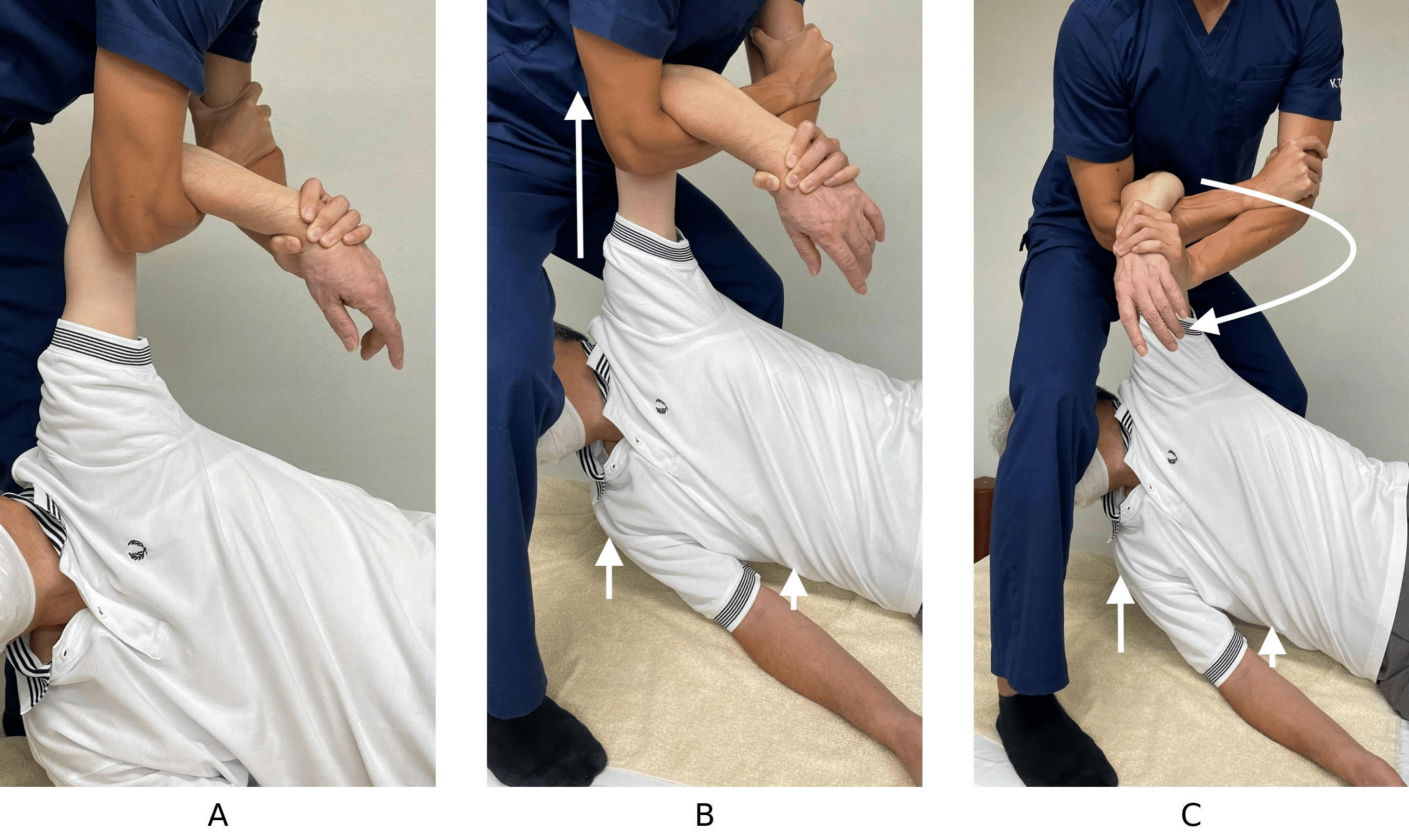
Sequential images demonstrating the lateral position maneuver (LPM) for anterior shoulder dislocation reduction. A: The patient’s shoulder is gently abducted, and the elbow is flexed. The clinician inserts their arm beneath the patient’s elbow. It is recommended to place a towel or similar padding between the arms to enhance stability. B: With shoulder abduction maintained, upward traction is applied slowly until the patient’s upper torso lifts slightly from the bed. The patient is encouraged to take deep breaths during this phase. C: While maintaining traction, the shoulder is gradually externally rotated to facilitate reduction.

A demonstration video (Video 1) and images (Figure 1) were created to illustrate the steps of the LPM technique. Co-author Dr. Nobuhiko Makihara acted as the simulated patient in the video. The shoulder was not dislocated, and the maneuver was performed for educational purposes only. Written informed consent for participation and publication was obtained.

**Video 1:** LPM Method Procedure Demonstration of the lateral position maneuver (LPM) by the authors. This is an original educational video created by the authors to demonstrate the LPM technique. The individual acting as the patient is co-author Dr. Nobuhiko Makihara. No actual shoulder dislocation was present. The demonstration was conducted on a healthy shoulder to illustrate each procedural step. He provided written informed consent for his participation and for publication of this video.

Clinical evaluation

Demographics, body mass index (BMI), prior dislocation history, affected side, presence of fractures or Bankart lesions, number of reduction attempts, and complications were recorded. For diagnostic evaluation, anteroposterior and lateral radiographs along with a computed tomography (CT) scan were performed. Post-reduction confirmation was done using anteroposterior and lateral radiographs.

## Results

Patient demographics and clinical characteristics

Thirteen patients with anterior shoulder dislocation, including seven men (53.8%) and six women (46.2%), were included in this study. The mean age was 62.3 years, ranging from 35 to 90 years, and the mean body mass index (BMI) was 24.3 kg/m², with a range of 14.5 to 33.6 kg/m². The right shoulder was affected in eight patients (61.5%), while the left in five (38.5%). Among these patients, three had a prior history of shoulder dislocation, two had a concomitant greater tuberosity fracture, and three had a concomitant Bankart lesion. Detailed individual patient characteristics, including age, sex, BMI, prior dislocation history, the number of reduction attempts, and associated findings, are summarized in Table 1.

**Table 1:** The detail of cases All 13 enrolled patients successfully underwent reduction of shoulder dislocation using the LPM Method. F: female; M: male.

Patient	Sex	Age	Height(m)	Weight(kg)	Body mass index (kg/m^2^)	Affected side	History of shoulder dislocation	Number of repositioning	Complications of shoulder
No.									
1	F	75	1.5	58	25.8	Right	Yes	1	Bankart lesion
2	M	35	1.8	74.1	22.9	Right	Yes	1	No
3	M	38	1.78	75.2	23.7	Left	No	2	No
4	F	90	1.42	49	24.2	Right	No	1	No
5	F	83	1.51	33	14.5	Right	No	1	Greater tuberosity fracture
6	F	72	1.6	50	19.5	Left	No	1	No
7	M	49	1.67	85.2	30.6	Left	No	2	No
8	M	50	1.68	68.9	24.6	Left	Yes	1	No
9	M	50	1.67	58	20.9	Right	No	1	Greater tuberosity fracture
10	F	89	1.48	65	29.7	Left	No	1	Bankart lesion
11	F	79	1.49	74.8	33.6	Right	No	1	No
12	M	90	1.53	53.2	22.7	Right	No	1	Bankart lesion
13	M	70	1.75	84	27.4	Right	No	1	No

Reduction outcomes and safety with the LPM technique

A total of 85 patients with anterior shoulder dislocation were initially assessed for eligibility. After applying the exclusion criteria, including multiple trauma, the use of other reduction methods, or missing data, 72 patients were excluded, and 13 patients were included in the final analysis and treated using the LPM. Only patients whose shoulder dislocation was reduced by the lead author using the LPM technique were included in this study. At our institution, multiple reduction techniques are routinely employed, and different physicians adopt their preferred methods. Importantly, the vast majority of the 72 excluded cases were not omitted due to unsuitability for the LPM itself, but rather because they had already undergone successful reduction using other techniques performed by different clinicians prior to evaluation by the lead author. Therefore, these cases were not eligible for inclusion in this analysis, which was specifically designed to assess outcomes of the LPM technique as performed by a single operator. Among the 13 patients who underwent LPM, successful reduction was achieved on the first attempt in 11 patients (84.6%), while two patients (15.4%) required multiple attempts. No cases required conversion to other reduction techniques or procedural sedation, and no complications such as fractures or neurological injuries were reported. The inclusion process and reduction outcomes are summarized in Figure 1.

**Figure 2:**
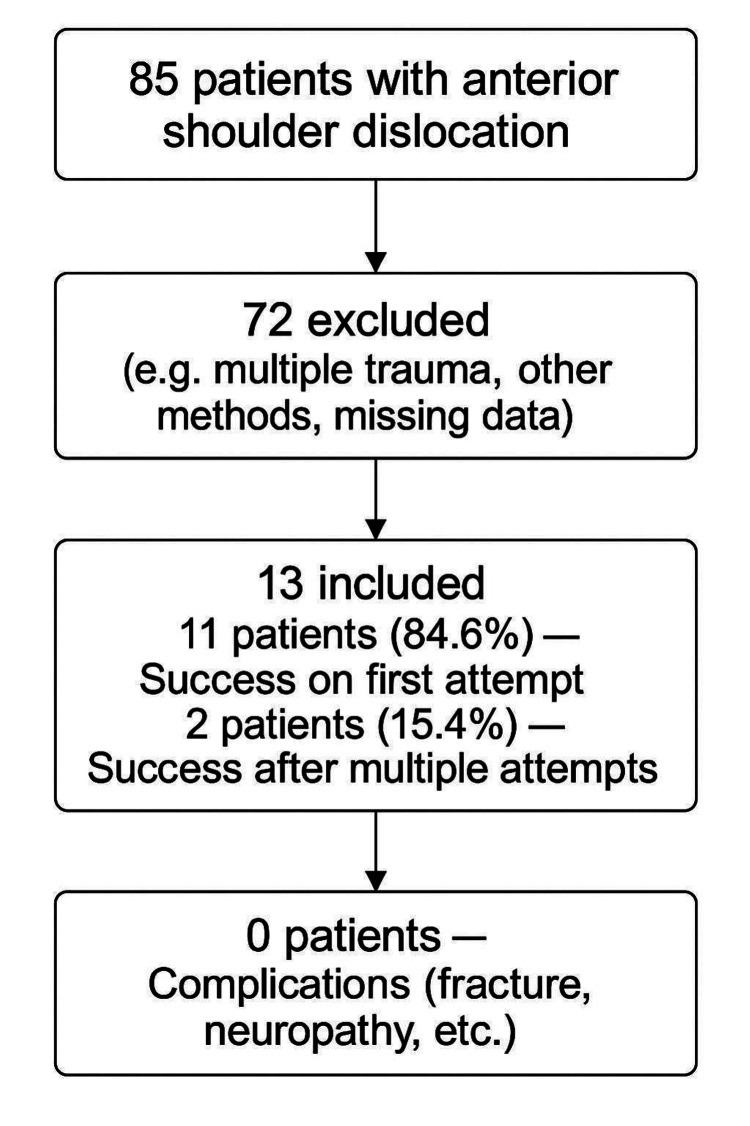
Flowchart of Patient Inclusion and Reduction Outcomes in This Study When Using the Lateral Position Maneuver.

## Discussion

Numerous reduction techniques for the anterior glenohumeral joint have shown varying effectiveness across published studies [2,5,7,9,13,14]. According to these reports, the success rate for first-time anterior shoulder dislocations ranges from 70% to 90%. Kuhn highlighted the paucity of data available to identify the most effective method for reducing shoulder dislocation [15], with 5% to 10% of cases proving irreducible in the emergency room [13,16]. Unlike most existing techniques, our demonstrated technique does not require assistance and sedation during the procedure [17]. We demonstrated that our technique is easy to perform as we were able to achieve a closed reduction of an acute anterior dislocation, requiring only a single physician without the use of sedation.

The lateral decubitus position could leverage gravity and body weight to apply sufficient traction on the dislocated joints. In the LPM, traction is applied upward to the point where the upper body is lifted. Thus, the LPM has a high likelihood of allowing the application of traction forces exceeding five pounds, which is the load required in the Stimson method [18], depending on the patient’s body weight and muscle mass. Although it has been reported that the success rate of the Milch procedure varies based on the presence or absence of a greater tuberosity fracture and that it is also more painful before and after the repositioning procedure [19], our method was shown to be useful for patients with large tuberosity fractures. In cases complicated by a greater tuberosity fracture, the accompanying pain may likely complicate repair and lead to increased muscle resistance. The LPM method enables a gradual increase of traction force, minimizing complaints of severe pain. According to Cunningham, shoulder reduction using the external rotation method typically occurs between 70° and 90° of external rotation [20]. In our series, although LPM allows for external rotation up to 90°, all reductions were successfully achieved at angles less than 90°. Moreover, a single surgeon can perform abduction and external rotation while applying a certain amount of traction force and can maintain a limb position that is advantageous for reduction.

Heavier patients (such as those in the current study who required two applications of the LPM technique to achieve reduction) are expected to require greater traction forces. The surgeon must be strong enough to maintain traction and slowly apply external rotation in such patients. Surgeons with smaller physical builds may encounter challenges performing the LPM technique on heavier patients.

Although five of the 13 patients in this study were aged 50 or younger, the overall sample was somewhat skewed toward older individuals. This age distribution may have contributed to the high success rate, as elderly patients often have more relaxed musculature, which can facilitate reduction. However, it is worth noting that the technique was also successful in two younger patients (aged 35 and 38) with a BMI over 22, suggesting that the maneuver may be feasible even in individuals with relatively higher muscle tone. Nonetheless, further data are needed to determine whether the technique is equally effective in broader younger populations, where single-operator reduction without anesthesia may be more challenging. Additionally, this study was conducted at a single institution by a single operator, and the sample size was relatively small. These factors limit the generalizability of our findings. Furthermore, because of insufficient data regarding the specific reduction techniques and the number of attempts used by other physicians in the excluded cases, direct comparisons with other established methods could not be performed, making it difficult to draw conclusions regarding the superiority of the lateral position maneuver. Nevertheless, this method may represent a useful alternative in specific clinical contexts, particularly where sedation or assistance is not readily available.

## Conclusions

In this case series, we introduced and evaluated a novel method for the reduction of anterior shoulder dislocations - the lateral position maneuver (LPM, Makihara method). Successful closed reduction was achieved in all 13 cases without sedation, assistance, or specialized equipment, including patients with complicating factors such as greater tuberosity fractures and recurrent dislocations. The first-attempt success rate was 84.6%, comparable to the existing techniques. Although a direct comparison was not performed, the LPM may serve as a simple and effective alternative, especially in resource-limited settings. Limitations include a small sample size, a single-center design, and a predominance of older patients, which may have influenced outcomes. Larger prospective studies are needed to validate the generalizability and effectiveness of this technique.
